# The mediating role of psychological inflexibility and social avoidance in relationship between body image disturbance and depression among young adults with acne in China

**DOI:** 10.3389/fpubh.2026.1771242

**Published:** 2026-03-10

**Authors:** Fen Xu, Mei Chan Chong, Yunxian Zhou, Junya Chen, Jiayin Ruan, Yue Sun, Juping Tang, Nor Aziyan Yahaya

**Affiliations:** 1 Department of Nursing Science, Faculty of Medicine, University of Malaya, Kuala Lumpur, Malaysia; 2 Department of Dermatology, Hangzhou Third Hospital, Hangzhou, China; 3 School of Nursing, Zhejiang Chinese Medical University, Hangzhou, China; 4 Rory Meyers College of Nursing, New York University, New York, NY, United States; 5 Nursing Department, Hangzhou Third Hospital, Hangzhou, China

**Keywords:** acne, body image disturbance, depression, mediation, psychological inflexibility, social avoidance

## Abstract

**Background:**

Acne impairs physical appearance and triggers significant psychological distress, particularly depression, in young adults, but the underlying psychological mechanisms remain underexplored.

**Objectives:**

This study aimed to examine the potential mediating associations of psychological inflexibility and social avoidance in the relationship between body image disturbance and depressive tendencies among young adults with acne in East China, based on the Acceptance and Commitment Therapy (ACT) framework.

**Methods:**

A cross-sectional survey was conducted among 201 young adults (18–29 years, 72.1% female) with acne from two tertiary hospitals in East China between December 2024 and February 2025. Validated instruments were used to assess body image disturbance (BIDQ), psychological inflexibility (AAQ-II), social avoidance (SADS-SA), and depression (PHQ-9). A serial mediation model (PROCESS Model 6) was analyzed via SPSS 29 with 5,000 bootstrap samples.

**Results:**

Correlation analysis showed significant positive relationships between body image disturbance, psychological inflexibility, social avoidance, and depression (all *p* < 0.01). Mediation analysis revealed that body image disturbance had a significant total effect on depression [Effect = 0.147, 95% CI (0.088, 0.205)], while its direct effect was non-significant. Two indirect pathways accounted for 84.1% of the total effect: (1) a single mediation through psychological inflexibility (70.8% of the effect); and (2) a serial mediation through psychological inflexibility and social avoidance (7.4% of the effect). Additionally, acne severity significantly predicted psychological inflexibility (*β* = 0.184, *p* = 0.012).

**Conclusion:**

Body image disturbance indirectly influences depression primarily through the pathways of psychological inflexibility and social avoidance. Integrating ACT into dermatological care to enhance psychological flexibility may effectively alleviate depressive symptoms in acne patients.

## Plain Language Summary

Acne is very common among young adults in China, but it is more than just a skin problem. It often makes people feel insecure about their appearance (called “body image disturbance”) and even is associated with depression. We wanted to understand the pathway connecting poor body image and depression in people with acne.

We studied 201 young adults (aged 18–29) with acne from two hospitals in Hangzhou. They completed questionnaires assessing their body image, “psychological inflexibility” (how much they get stuck in negative thoughts/feelings, such as avoiding unpleasant emotions), social avoidance (missing social events to hide acne), and depressive symptoms. We then examined the links between these factors.

Here’s what we found: Poor body image does not cause depression directly. Instead, two indirect pathways explain this connection:

The main pathway: Poor body image is associated with increased psychologically inflexibility, individuals may fixate on thoughts like “I look ugly because of acne” and avoid uncomfortable feelings. This inflexibility is ultimately linked to higher levels of depression.

A smaller, secondary pathway: Poor body image first makes people inflexible, which is linked to avoid social situations (e.g., refusing to attend parties). This avoidance is associated with an increased risk of depression.Importantly, social avoidance alone does not cause depression—it only has an impact after psychological inflexibility occurs.

Care recommendations: Doctors and nurses should screen acne patients for psychological inflexibility during consultations using a simple tool called the AAQ-II. Adding short, effective mental health interventions (such as mindfulness exercises or discussions with patients about what matters to them) to acne treatment can help patients improve their psychological flexibility. This way, we can treat both skin and mental health issues, thereby enhancing overall well-being.

Future studies should follow patients over the long term, include more people from different regions of China, and could also expand to adolescents or older adults to verify these results.

## Highlights

- This research found that psychological inflexibility is a major mediating factor between body image disturbance and depressive symptoms in young acne patients, supporting the theoretical framework of Acceptance and Commitment Therapy (ACT).
- The results of this study reveal a significant chain-mediating effect between psychological rigidity and the resulting social avoidance, providing new insights into the cognitive and behavioral pathways of depression.
- Based on the findings of this study, it is recommended that ACT-based psychological interventions be integrated into dermatological treatment, providing intervention directions for addressing the potential mental health problems of acne patients. The findings highlight the clinical value of integrating ACT-based psychological interventions into dermatology care to address underlying mental health issues in patients with acne.

## 1 Introduction

Acne ranks among the most prevalent dermatological conditions that impact adolescents and young adults, with a global prevalence exceeding 85% in this age group (1). While the physical manifestations of acne are well-documented, recent research has increasingly highlighted its psychosocial consequences, including impaired self-esteem, social withdrawal, and elevated rates of anxiety and depression (2, 3). In the Chinese context, acne is not only recognized as a medical issue but also a visible indicator of perceived imperfection (4). Notably, under the influence of appearance-centered social media trends, this perception may further exacerbate psychological distress among affected individuals (5). A population-based study of Chinese college students confirmed a high prevalence of acne and demonstrated significant impairment in quality of life, emphasizing the psychosocial burden that extends beyond physical symptoms (4).

A core psychological consequence of acne is body image disturbance is characterized by persistent negative self-perception and dissatisfaction with physical appearance (6). Previou studies have established a robust association between body image concerns and depression in young adults, specifically, greater degrees of body image dissatisfaction show a notable correlation with an increased likelihood of experiencing depressive symptoms (7, 8). In China, similar findings have been reported, with body dissatisfaction identified as a key predictor of mental health problems in university populations (9). However, the mechanisms underlying this association remain underexplored.

The Acceptance and Commitment Therapy (ACT) model provides a robust framework for understanding how intrinsic cognitive factors particularly psychological inflexibility that underlie psychological disorders (10). In ACT, psychological inflexibility is defined as a mental state characterized by cognitive fusion and experiential avoidance, which leads to a disconnection between behaviors and core values and entraps individuals in rigid coping patterns (10, 11). Its essence lies in the lack of adaptive psychological regulation capabilities based on awareness and acceptance. Investigations have proven that psychological inflexibility functions as a mediator between negative self-cognition and psychological problems such as depression (12). In patients with skin diseases, psychological inflexibility may amplify the emotional responses caused by body image disturbance; however, this mediating pathway remains lacking in empirical validation.

Social avoidance represents a common behavioral response to appearance-related distress., individuals with visible skin conditions frequently exhibit interpersonal avoidance driven by evaluation fears.” (13). Acne patients may encounter heightened social anxiety, that leading to avoidance behaviors (14). Previous research demonstrated that social avoidance can lead to reduced social support, exacerbate feelings of loneliness and depression, and thus form a vicious cycle (15). From the perspective of Chinese cultural background, where collectivism and the concept of “face” (mianzi) are highly valued, appearance-related social anxiety may be particularly pronounced (16).

Although several studies have independently linked body image disturbance to psychological inflexibility (17), social avoidance (15), and depression (9, 18, 19), few have examined these variables within an integrated framework. Emerging research suggests that these factors may function in a sequential pathway, where body image disturbance fosters psychological inflexibility, which leads to social avoidance, ultimately increasing vulnerability to depression (20). However, to date, no study has systematically tested this chain mediation model in a Chinese acne population.

The present study addressed this research gap by investigating the sequential mediating effects of psychological inflexibility and social avoidance on the relationship between body image disturbance and depression in young adults with acne in East China. Through clarifying these pathways, the present study sought to enhance understanding of acne’s psychological impact and to provide insights for targeted interventions that address both internal cognitive processes and external social behaviors.

## 2 Methods

### 2.1 Design and participants of the study

This study adopted a cross-sectional survey design, with participants recruited via consecutive sampling. Young adult patients diagnosed with acne who visited the dermatology outpatient departments of two hospitals in Hangzhou, Zhejiang Province, China, constituted the study sample. Specifically, both participating hospitals are tertiary-level hospitals specializing in dermatology, and they admit dermatology patients from various areas in eastern China. Data collection was conducted from December 2024 to February 2025, during which standardized data collection protocols were strictly followed to ensure the accuracy and completeness of the collected information.

The implementation of this study obtained formal ethical approval from the Institutional Review Board (IRB). At their first clinical visit, eligible participants received a written informed consent form, supplemented with verbal explanations to guarantee their understanding, all participants were fully informed of the study’s background, objectives, and procedures, and written informed consent was voluntarily provided by each participant. Only participants who submitted written informed consent were admitted to the research. Throughout the entire research process, all data collection methods and participant rights protection measures strictly complied with the requirements outlined in the latest revision of the Declaration of Helsinki, ensuring the ethical integrity of the study.

The inclusion criteria were as follows: (1) meeting the diagnostic criteria for acne vulgaris as specified in the Guidelines for the Diagnosis and Management of Acne Vulgaris in Primary Care Settings (21); (2) aged between 18 and 29 years; (3) acne duration of no less than 3 months; (4) unimpaired consciousness without cognitive disorders or comprehension difficulties; (5) being willing to provide written informed consent. Meanwhile, participants were removed from the study if they: (1) had received structured psychological interventions from psychiatrists or psychologists; (2) suffered from cognitive deficits or psychiatric conditions [psychiatric screening was performed by a senior dermatologist via clinical interview based on DSM-5 diagnostic criteria (22)] that would prevent completion of the study; or (3) suffered from other severe skin diseases or chronic systemic illnesses.

GPower 3.1 software was utilized to calculate the sample size required for this study. Referring to prior similar studies on body image disturbance, psychological inflexibility, and depression in dermatological populations, A medium effect size (specified as *f*^2^ = 0.15) was employed in the calculation. Meanwhile, the significance level (*α*) was fixed at 0.05, and the statistical power (1-*β*) was set to 0.80, and considering 1 independent variable (body image disturbance), 2 mediating variables (psychological inflexibility, social avoidance), and 1 dependent variable (depression), the calculated minimum sample size was 180. To account for potential non-response or missing data, an additional 10% of participants were recruited.

### 2.2 Data collection

The questionnaire survey in this study was conducted by three nurses with postgraduate education backgrounds. Prior to the initiation of data collection, all members of the research team received standardized training, which covered content such as familiarization with the questionnaire items, methods for communicating with physicians, strategies for effective communication with patients, and consistent approaches to answering patients’ inquiries—all aimed at ensuring the homogenization principle of the study and maintaining consistency in data collection.

Validated instruments were used for data collection, with prior permission obtained from the original authors. The following tools were employed:

#### 2.2.1 Body image disturbance questionnaire

This is a standardized self-report scale designed to assess body image-related issues in individuals. It is widely applied in fields such as psychology, dermatology, and plastic surgery, with a specific focus on measuring individuals’ negative cognition toward their own appearance, emotional responses, and related behavioral tendencies (23). Comprising 7 items, the scale covers dimensions including appearance-related concerns, emotional distress, social/occupational impacts, and avoidance behaviors. A Likert-scale scoring method is adopted, where higher scores indicate more severe body image disturbance (23). The Chinese version of the BIDQ, revised by Chen et al., has demonstrated a Cronbach’s *α* coefficient of 0.82 (24). In the present study, its Cronbach’s α coefficient was 0.89, indicating good reliability consistent with the validated version.

#### 2.2.2 Social avoidance and distress scale

This 28-item scale is specifically designed to assess social anxiety and social avoidance levels (25), focusing on social interaction avoidance tendencies. For the SADS, the range of total scores is 0 to 28, where higher scores denote more significant degrees of social avoidance and emotional distress. In this study, the social avoidance subscale (SADS-SA) was used, with scores ranging from 0 to 14. The Chinese adaptation of the SADS has exhibited a Cronbach’s *α* coefficient of 0.85, with reliability coefficients of 0.77 for the avoidance subscale and 0.73 for the distress subscale (26). In the current study, the Cronbach’s α coefficient for the SADS-SA subscale was 0.83.

#### 2.2.3 Acceptance and action questionnaire-II

AAQ-II is a self-report scale designed to assess psychological flexibility, specifically measuring an individual’s ability to accept emotional and cognitive distress and behave in accordance with their values despite these difficulties (27). The scale consists of 7 items, with responses rated on a 7-point Likert scale ranging from “1 - Never true” to “7 - Always true.” Higher scores indicate greater psychological inflexibility and experiential avoidance. The Chinese version of the AAQ-II has been validated and demonstrates strong reliability and validity. It shows high internal consistency, with a Cronbach’s *α* of 0.88, and has been confirmed for structural validity through confirmatory factor analysis (28). In this study, the Cronbach’s α for the AAQ-II was 0.94.

#### 2.2.4 Patient health questionnaire-9

The PHQ-9 is a widely used 9-item self-report scale designed to screen for, diagnose, and assess the severity of depression. Respondents are asked to rate the frequency of specific depressive symptoms experienced over the past 2 weeks (29). The responses are measured on a 4-point scale, ranging from “0 - Not at all” to “3 - Nearly every day”. The total score ranges from 0 to 27, with higher scores reflecting greater self-reported severity of depression. The PHQ-9 has demonstrated strong psychometric properties, including high reliability and validity across different populations. The original version shows excellent internal consistency, with a Cronbach’s alpha of 0.89, and has demonstrated good criterion validity. Additionally, the scale has been translated and validated in multiple languages, with the Chinese version, validated by Wang et al., showing strong reliability (Cronbach’s alpha = 0.88) and confirmed validity through exploratory factor analysis (30). In clinical practice, the PHQ-9 is not only used as a screening tool but also to monitor changes in the severity of depression, making it an indispensable instrument for both diagnosis and treatment evaluation. In this study, the PHQ-9 was employed to assess the severity of depression among participants, and the Cronbach’s alpha for this scale in the present study was 0.92.

Regarding data quality control, 19 invalid questionnaires were excluded from the initial sample (*n* = 220), resulting in a final analytical sample of 201 participants. The exclusions were based on the following criteria: response time per item less than 2 s (*n* = 6), patterned or repetitive responses (*n* = 8), and missing key variables (*n* = 5).

### 2.3 Data analysis

Statistical analyses were performed via SPSS 29.0 and PROCESS macro 3.5. Preliminary analyses included Descriptive statistics and bivariate correlations to examine variable distributions and associations. A serial mediation model (Model 6 in PROCESS), as suggested by Hayes (31), was applied to test the hypothesized mediation pathways. The model specified: Body image disturbance was entered as the independent variable (X), depression as the dependent variable (Y), psychological inflexibility as the first mediator (M1), and social avoidance as the second mediator (M2). All variables were mean-centered before analysis. The significance of indirect effects was assessed through 5,000 bootstrap resamples and 95% bias-corrected confidence intervals; effects were considered statistically significant if the confidence intervals(CIs) excluded zero. To control for potential confounders, age, gender, acne grading, and duration were included as covariates in the mediation analysis. Multicollinearity was assessed using Variance Inflation Factor (VIF).

## 3 Results

### 3.1 Descriptive statistics

Demographically, most were aged 25–29 years (62.2%), female (72.1%), urban residents (71.6%), and had a college education (72.1%); over three-quarters (78.1%) had a monthly household income of ≥5,000 CNY, and 67.7% were unmarried. Clinically, acne duration was mostly 1–3 years (33.8%), with lesions primarily limited to the face and neck (74.1%); 42.8% had moderate acne, 26.4% had mild acne, 22.9% had moderately severe acne, and 8.0% had severe acne, and 28.9% had a family history of acne. For treatment (patients may have used multiple modalities), external therapy (topical application/light/laser, 69.7%) and combined Chinese-Western medicine (53.7%) were the most frequently used (see Table 1).

**Table 1:** Demographic and clinical characteristics (*N* = 201).

Variables	Number (*n*)	Proportion (%)
Age (year)		
18–24	76	37.8
25–29	125	62.2
Gender		
Male	56	27.9
Female	145	72.1
Marital status		
Unmarried	136	67.7
Married	62	30.8
Divorced	3	1.5
Residence		
Urban	144	71.6
Rural	33	16.4
Town	24	11.9
Educational level		
Junior school and below	10	5.0
Senior school	25	12.4
College	145	72.1
Master and above	21	10.5
Medical insurance		
Employee medical insurance	107	53.2
Resident medical insurance	42	20.9
Student medical insurance	30	14.9
Self-paying/others	22	11.0
Working status		
Students	49	24.4
Unemployed	8	4.0
Clerks	111	55.2
Individual businesses	22	10.9
Workers	11	5.5
Monthly household income (CNY ¥)		
< 3,000	8	4.0
3,000–4,999	36	17.9
5,000 and above	157	78.1
BMI (kg/m^**2**^)		
< 18.5	27	13.4
18.5–23.9	143	71.1
24 and above	31	15.4
Duration of acne (years)		
< 1	18	9.0
1–3	68	33.8
4–6	47	23.4
7–10	35	17.4
10 and above	33	16.4
Site of acne		
Face and neck	149	74.1
The face, neck, and torso	46	22.9
Torso only	6	3.0
Acne grading		
Mild	53	26.4
Moderate	86	42.8
Moderately severe	46	22.9
Severe	16	8.0
Oral traditional Chinese medicine only		
No	164	81.6
Yes	37	18.4
Oral Western medicine only		
No	139	69.2
Yes	62	30.8
Combination of Chinese medicine and Western medicine		
No	93	46.3
Yes	108	53.7
External treatment (topical application, external application, and light/laser therapy)		
No	61	30.3
Yes	140	69.7
Family history of acne		
No	143	71.1
Yes	58	28.9

Acne grading was assessed by one attending physician or above with extensive clinical experience using the Pillsbury grading system.

### 3.2 Correlation analysis

Pearson correlation analysis demonstrated significant positive associations among all study variables (*p* < 0 0.01). Specifically: Body image disturbance showed strong correlations with psychological inflexibility, social avoidance, and depression. Psychological inflexibility and social avoidance were also significantly interrelated and correlated with depression (see Table 2).

**Table 2:** Correlation variables with Pearson’s Correlation Coefficient.

Variables	Body image disturbance (BIDQ)	Psychological inflexibility (AAQ-II)	Social avoidance (SADS-SA)	Depression (PHQ-9)
Body image disturbance (BIDQ)	–	0.389**	0.343**	0.370**
Psychological inflexibility (AAQ-II)	0.389**	–	0.568**	0.767**
Social avoidance (SADS-SA)	0.343**	0.568**	–	0.540**
Depression (PHQ-9)	0.370**	0.767**	0.540**	–

**p < 0.01.

### 3.3 Multicollinearity test

As shown in Table 3, the three predictors had tolerance values of 0.635–0.827 and VI*F* values of 1.210–1.576, all within acceptable ranges (Tolerance > 0.1, VIF < 5). This confirms no significant multicollinearity, ensuring reliable regression and mediation results.

**Table 3:** Results of multicollinearity test.

Variable name	Tolerance	VIF
Depression (PHQ-9)	–	–
Body image disturbance (BIDQ)	0.827	1.210
Psychological inflexibility (AAQ-II)	0.635	1.576
Social avoidance (SADS-SA)	0.660	1.515

### 3.4 Regression analysis of the mediation model

As shown in Table 4, the regression model for psychological inflexibility (M1) was significant (*F* = 7.523, *p* < 0.001), explaining 18.9% of its variance. Body image disturbance (X) (*β* = 0.360, *p* < 0.001) and acne severity (*β* = 0.184, *p* = 0.012) were positively associated with psychological inflexibility, while other covariates showed no significant associations (all *p* > 0.05).

**Table 4:** Regression coefficients of the covariate-adjusted serial mediation model (*N* = 201).

Dependent variable	Predictor variable	Unstandardized coefficient (B)	Standard error (SE)	Standardized coefficient (β)	*t*	*p*	95% Confidence interval
Psychological inflexibility (M1)	Constant	7.368	4.934	–	1.494	0.137	[−2.362, 17.099]
	Body image disturbance (X)	0.270	0.050	0.360	5.390	<0.001***	[0.171, 0.369]
	Gender	2.015	1.533	0.097	1.315	0.190	[−1.007, 5.038]
	Age	−1.022	1.277	−0.054	−0.800	0.425	[−3.541, 1.497]
	Acne grading	1.928	0.764	0.184	2.524	0.012*	[0.422, 3.435]
	Acne duration	−0.088	0.507	−0.012	−0.174	0.862	[−1.089, 0.912]
	BMI	−1.208	1.148	−0.069	−1.052	0.294	[−3.472, 1.056]
	Model fit	*R* = 0.435, *R*^2^ = 0.189, *F* = 7.523, *p* < 0.001					
Social avoidance (M2)	Constant	0.227	1.810	-	0.125	0.901	[−3.343, 3.796]
	Body image disturbance (X)	0.043	0.020	0.142	2.200	0.029*	[0.005, 0.082]
	Psychological inflexibility (M1)	0.203	0.026	0.501	7.748	<0.001***	[0.151, 0.255]
	Gender	0.558	0.561	0.066	0.994	0.321	[−0.549, 1.666]
	Age	−0.308	0.467	−0.040	−0.660	0.510	[−1.228, 0.612]
	Acne severity	0.125	0.283	0.030	0.443	0.658	[−0.433, 0.684]
	Acne duration	−0.061	0.185	−0.020	−0.327	0.744	[−0.426, 0.304]
	BMI	−0.131	0.420	−0.019	−0.312	0.755	[−0.959, 0.697]
	Model fit	*R* = 0.588, *R*^2^ = 0.346, *F* = 14.585, *p* < 0.001					
Depression (Y)	Constant	−3.931	2.004	-	−1.961	0.051	[−7.885, 0.022]
	Body image disturbance (X)	0.023	0.022	0.053	1.062	0.290	[−0.020, 0.067]
	Psychological inflexibility (M1)	0.384	0.033	0.658	11.553	<0.001***	[0.318, 0.449]
	Social avoidance (M2)	0.199	0.080	0.139	2.504	0.013*	[0.042, 0.357]
	Gender	0.523	0.623	0.043	0.839	0.403	[−0.707, 1.753]
	Age	0.507	0.517	0.046	0.981	0.328	[−0.513, 1.528]
	Acne grading	0.165	0.314	0.027	0.527	0.599	[−0.454, 0.784]
	Acne duration	−0.225	0.205	−0.051	−1.099	0.273	[−0.630, 0.179]
	BMI	−0.375	0.465	−0.037	−0.805	0.422	[−1.292, 0.543]
	Model fit	*R* = 0.784, *R*^2^ = 0.615, *F* = 38.263, *p* < 0.001					

*p < 0.05, **p < 0.01, ***p < 0.001, β: standardized coefficient; Covariates include:gender, age, acne grading, acne duration, and BMI; 4. Model fit indices (R, R^2^, F value) are provided for each outcome variable to demonstrate model adequacy.

The social avoidance (M2) model was also significant (*F* = 14.585, *p* < 0.001) (*R*^2^ = 34.6%). Both body image disturbance (*β* = 0.142, *p* = 0.029) and psychological inflexibility (*β* = 0.501, *p* < 0.001) were positively linked to social avoidance, with no significant covariate effects (all *p* > 0.05).

The depressive symptoms (Y) model fit well (*F* = 38.263, *p* < 0.001, *R*^2^ = 61.5%). Psychological inflexibility (*β* = 0.658, p < 0.001) and social avoidance (*β* = 0.139, *p* = 0.013) positively predicted depression, whereas the direct effect of body image disturbance was non-significant (*β* = 0.053, *p* = 0.290), indicating substantial statistical mediation. Covariates had no significant impacts on depression (all *p* > 0.05).

### 3.5 Bootstrap indirect effect analysis

Table 4 presents the results of bootstrap indirect effect analysis with 5,000 resamples. The total effect of body image disturbance on depressive symptoms was significant [Effect = 0.147, 95% CI (0.088, 0.205)]. The total indirect effect was 0.123 [95% CI (0.072, 0.177)], accounting for 84.1% of the total effect, confirming that the association between body image disturbance and depression was primarily mediated by the proposed pathways.

Two significant indirect pathways were identified: (1) The single mediation pathway through psychological inflexibility (X → M1 → Y) [Effect = 0.104, 95% CI (0.053, 0.161)] accounted for 70.8% of the total effect, serving as the dominant pathway. ② The serial mediation pathway through psychological inflexibility and social avoidance (X → M1 → M2 → Y) [Effect = 0.011, 95% CI (0.001, 0.022)] accounted for 7.4% of the total effect. The single pathway through social avoidance (X → M2 → Y) was non-significant [Effect = 0.009, 95% CI (−0.001, 0.022)].

Effect comparisons showed the dominant pathway (X → M1 → Y) had a significantly larger effect than the other two pathways (both *p* < 0.05), with no significant difference between Path 2 and Path 3 (*p* > 0.05). All mediation effects reflect statistical associations rather than causal relationships due to the cross-sectional design (Table 5).

**Table 5:** Bootstrap indirect effects (5,000 samples, bias-corrected).

Effect type	Effect value	Bootstrap SE	95% Confidence interval	Percentage of total effect (%)	Significance
Total effect (X → Y)	0.147	0.030	[0.088, 0.205]	100.0	✓
Direct effect (X → Y)	0.023	0.022	[−0.020, 0.067]	15.9	X
Total indirect effect	0.123	0.027	[0.072, 0.177]	84.1	✓
Indirect path 1 (X → M1 → Y)	0.104	0.028	[0.053, 0.161]	70.8	✓
Indirect path 2 (X → M2 → Y)	0.009	0.006	[−0.001, 0.022]	5.9	X
Indirect path 3 (X → M1 → M2 → Y)	0.011	0.005	[0.001, 0.022]	7.4	✓
Effect comparison 1 (Path1-Path2)	0.095	0.029	[0.041, 0.154]	–	✓
Effect comparison 2 (Path1-Path3)	0.093	0.029	[0.039, 0.149]	–	✓
Effect comparison 3 (Path2-Path3)	−0.002	0.005	[−0.013, 0.007]	–	X

Significance: ✓ = Significant (95% CI excludes 0); X = Non-significant (95% CI includes 0).

### 3.6 Summary of findings

**Figure 1:**
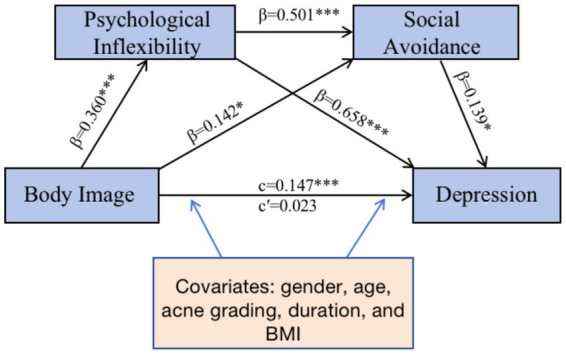
Chained mediation model. *p < 0.05; ***p < 0.001 β: Standardized coefficient.

The analysis revealed a serial mediation pathway connecting body image disturbance to depressive symptoms through psychological inflexibility and social avoidance (Figure 1). Body image disturbance demonstrated a strong predictive relationship with psychological inflexibility (*β* = 0.360, *p* < 0.001), which in turn significantly predicted both social avoidance (*β* = 0.501, *p* < 0.001) and depressive symptoms (*β* = 0.658, *p* < 0.001). Social avoidance also contributed significantly to depressive symptoms (*β* = 0.139, *p* = 0.013).

A direct pathway between body image disturbance and social avoidance was also identified (*β* = 0.142, *p* = 0.029). The total effect of body image disturbance on depression (Effect = 0.147, *p* < 0.001) became non-significant when accounting for the mediators (direct effect c’ = 0.023, *p* = 0.290), indicating full statistical mediation.

The model accounted for 61.5% of variance in depressive symptoms (*R*^2^ = 0.615). Psychological inflexibility was the strongest mediator, with its indirect effect (Effect = 0.104) stronger than the chained pathway (Effect = 0.011).

## 4 Discussion

This study explored the psychological mechanisms underlying the association between body image disturbance and depressive symptoms in acne-affected young adults, with a focus on the mediating effects of psychological inflexibility and social avoidance. The findings revealed no significant direct effect of body-image distress on depression. Instead, two distinct indirect pathways emerged: (1) a significant mediation through psychological inflexibility alone, and (2) a sequential mediation involving psychological inflexibility followed by social avoidance. Notably, psychological inflexibility demonstrated complete mediation in the single-path model, establishing it as the predominant mechanism underlying the development of depressive symptoms.

In line with the Acceptance and Commitment Therapy (ACT) framework (32), our model explained 60.8% of the variance in depressive symptoms. While the direct effect of body image disturbance on depression was non-significant in this sample, we interpret this as a substantial indirect link rather than an exclusive one, acknowledging that unmeasured variables such as self-esteem or social support might also contribute to the remaining variance.

Body image disturbance, particularly the devaluation ofacne-affected appearance, may foster non-acceptance of bodily state, subsequently associated with features of psychological inflexibility such as cognitive fusion (“I am worthless”) and experiential avoidance (e.g., avoidance of social situations), which in turn exacerbate depressive symptoms (33). the current study’s results align closely with prior research: for instance, identified psychological inflexibility as a key predictor of depression, anxiety, and reduced subjective well-being across cultural and clinical contexts (27).

In the Chinese context, found that psychological inflexibility significantly mediated the relationship between body dissatisfaction and emotional distress in Chinese young adult population (34); similarly, a systematic review also reported a strong association between body-image distress and depression, often manifested via self-esteem, social support and perceived stress (19).

Prior research has seldom differentiated between the internal cognitive processes of psychological inflexibility and their external behavioral manifestations in social avoidance (35). The current findings advance this understanding by demonstrating their synergistic relationship, with psychological inflexibility emerging as the critical mediator linking body-image distress to depressive symptoms. These results imply that purely dermatological interventions targeting physical appearance may yield limited mental health benefits, whereas Acceptance and Commitment Therapy (ACT) specifically designed to enhance psychological flexibility, may offer more effective depression management in this population (36).

Social avoidance demonstrated no significant independent mediation effect, but contributed meaningfully within the sequential pathway. This pattern suggests avoidance behaviors primarily emerge as consequences of psychological inflexibility that manifested through eroded social confidence (“I cannot face others’ gaze”) rather than functioning as primary drivers of depression. The modest effect size (*β* = 0.013, 8% of total effect) aligns with conceptual models framing social avoidance as a secondary behavioral outcome of cognitive-emotional distress (37).

Moreover, cultural factors may moderate these mechanisms. In collectivist cultures like China, one’s body image is closely tied to social evaluation, and concerns about “losing face” or appearance-related shame (38) may amplify the impact of psychological inflexibility on social avoidance, thereby creating a culturally specific pathway to depression. This cultural amplification may explain why the chain mediation is significant yet small in magnitude, suggesting that cultural norms should be considered a key target for future interventions.

From a clinical nursing perspective, recommendations include the integration of psychological techniques with dermatological care. First, screen for high-risk individuals with psychological inflexibility using the AAQ-II at initial consultation, followed by one-on-one education or group workshops to correct rigid beliefs (e.g., “I must eliminate acne before life can proceed normally”) and foster adaptive mindsets; interventions enhancing psychological flexibility—emphasizing openness and active coping—alleviate shame in dermatology patients and support adaptive skin management behaviors. Second, integrate mindfulness-based cognitive therapy (MBCT) as an adjunct treatment for dermatology patients and embed brief ACT exercises—such as mindful sensory awareness during topical application—into routine care to help patients clarify core values, set small value-aligned goals, and track progress in value-driven behaviors (39, 40). Additionally, cognitive-behavioral therapy (CBT) has exhibited effectiveness in tackling psychological issues among dermatology patients by reducing stress, anxiety, and negative affect (41). Nurses, in collaboration with psychotherapists, can design “social exposure hierarchies” that gradually progress from low- to high-anxiety scenarios and establish community or campus support groups using role-play activities to diminish social evaluation fears (42). For severe cases, involving family support systems can further bolster social confidence (43). The proposed integrated care model combines dermatological nursing, psychological support, and community follow-up in a closed-loop system, enhanced by a mobile application providing mindfulness resources and progress-tracking features. This integrated approach enhances intervention accessibility and continuity, reorienting treatment goals from symptom control to improved quality of life. It addresses physical and psychological care dimensions via coordinated clinical and digital elements.

### 4.1 Limitations

Despite the meaningful findings, this study has several limitations that should be acknowledged. Given the cross-sectional nature of this study, we acknowledge that reverse causality cannot be ruled out (e.g., depressive symptoms may also influence body image disturbance and psychological inflexibility). We are unable to establish definitive causal directions among the variables, and longitudinal studies with repeated measurements are needed to verify the temporal sequence of the proposed pathway. Additionally, we acknowledge the conceptual overlap between psychological inflexibility (AAQ-II) and general distress (PHQ-9), as well as the potential overlap between the avoidance dimension of BIDQ and social avoidance (SADS-SA). Sensitivity analyses with covariate adjustment confirmed that the mediation pathways remain significant, indicating the robustness of the findings. Finally, our sample was recruited from two tertiary hospitals and was predominantly female, which may limit the generalizability of the findings to male patients or community-dwelling individuals with milder acne.

## 5 Conclusion

The findings from this study indicated that psychological inflexibility and social avoidance significantly mediated the relationship between body image disturbance and depression in young East Chinese adults with acne, accounting for a substantial portion of the total effect. These results support integrating Acceptance and Commitment Therapy principles particularly flexibility-enhancing strategies like mindfulness training along with conventional dermatological care. Future investigations utilizing longitudinal designs and expanded sampling would help establish causal relationships and enhance external validity.

## Data Availability

The raw data supporting the conclusions of this article will be made available by the authors, without undue reservation.
